# Optical coherence tomography and electrophysiological analysis of proptotic eyes due to thyroid-associated ophthalmopathy

**DOI:** 10.1007/s10792-022-02605-x

**Published:** 2022-12-08

**Authors:** Mahmoud Ahmed Elsamkary, Amany Abd El-Fattah El-Shazly, Tamer Abdel Fattah Badran, Yousef Ahmed Fouad, Randa Hesham Ali Abdelgawad

**Affiliations:** Faculty of Medicine, Ain Shams University, 11517 Ramses St. Abbassia, Cairo, Egypt

**Keywords:** Central foveal thickness, Pattern electroretinogram, Thyroid-associated ophthalmopathy, Optical coherence tomography

## Abstract

**Purpose:**

To study the degree of morphological and functional changes in thyroid-associated ophthalmology (TAO) patients using optical coherence tomography and electrophysiological studies and investigate their clinical correlation.

**Methods:**

A cross-sectional study including 40 patients with TAO and a control group with age- and sex-matched healthy subjects. All subjects underwent a complete ophthalmological examination, proptosis evaluation, spectral domain optical coherence tomography, and electrophysiological tests (pattern and multifocal electroretinograms, and visual evoked potentials).

**Results:**

On multiple regression analysis, the degree of proptosis and P50 amplitude were the most important predictors of central foveal thickness (CFT) (*β* =  −  0.56 and 0.39 and *p* = 0.03 and 0.01, respectively); while duration of the disease, and clinical activity score (CAS) were the most important predictors of average RNFL thickness (*β* = 0.67 and  − 0.81 and *p* = 0.004 and  <  0.001, respectively).

**Conclusion:**

In the absence of fundus changes, macular thinning along with functional alterations noticed by pattern and multifocal electroretinogram could indicate presence of subclinical retinopathy in TAO cases.

## Introduction

Thyroid-associated ophthalmopathy (TAO) is an autoimmune disorder characterized by infiltration of the orbital fat and interstitial tissues by inflammatory cells including lymphocytes, plasma cells, mast cells, and macrophages, and by retention of fluid and build-up of glycosaminoglycan. The ensuing expansion in the volume of orbital constituents results in increased intraorbital pressure [1, 2]. When involving the orbital apex, this can lead to compression of the optic nerve fibers (dysthyroid optic neuropathy, DON), which has been shown to be the most common cause of vision loss in TAO patients [3].

It has been demonstrated that DON can occur in TAO patients who do not experience extraocular muscle swelling [4–6]. Evaluation of visual function in TAO patients has been reported using various diagnostic and follow-up tests, including optical coherence tomography (OCT), visual field testing, visual evoked potential (VEP), and contrast sensitivity testing [7–9]. Especially appealing is the use of the noninvasive OCT to assess the structure of the macula and optic disk [8, 9]. Compared to healthy controls, TAO patients have been shown to have significantly thinner maculae and inferior retinal nerve fiber layer (RNFL) thicknesses [10].

Electrophysiological studies (EPS) that have been shown to provide objective evaluation of DON include VEP and pattern electroretinogram (PERG). Their results have also been shown to correlate with the clinical presentation and staging of the patients [11, 12]. VEP and PERG provide functional evaluation of the optic nerve and retinal ganglion cells [13]. Whether either is responsible for vision loss in TAO patients remains unclear. Therefore, in this study we aimed to investigate, using OCT, ERG and VEP, whether retinal or optic nerve dysfunction are the main pathology in TAO patients who experience visual impairment.

## Methods

### Patient selection

This cross-sectional study was conducted in the ophthalmology department of a tertiary care center during the 1-year interval of March 2018 to March 2019. We included the right eyes of 40 patients diagnosed with TAO with clinically apparent proptosis, in addition to the right eyes of 40 healthy individuals as controls.

We excluded patients with pathological myopia (spherical equivalent ≥  − 6 diopters and/or axial length [AL] ≥ 25 mm) or with a known retinal disorder such as diabetic retinopathy. Patients suffering from uveitis, drusen, glaucoma, previous eye trauma, retinal vascular abnormalities, age-related macular degeneration, opaque media, previous optic nerve disease or other chronic eye diseases were also excluded, as well as those with history of intraocular surgery, refractive surgery, intravitreal injection, or systemic diseases other than thyroid disease.

### Study procedures

All participants underwent full medical and ophthalmological history taking. Validation of the diagnosis of hyperthyroidism was by measuring serum levels of thyroid stimulating hormone (TSH), free triiodothyronine (T3), and thyroxine levels (T4). All subjects underwent ophthalmological assessment by best-corrected visual acuity (BCVA) measurement using Snellen chart (and transformed into logMAR), slit-lamp examination, Goldman applanation tonometry, indirect ophthalmoscopy, ocular motility examination, and evaluation of the proptosis using Hertel's exophthalmometer. The following seven criteria were used to calculate clinical activity score (CAS): spontaneous orbital pain, gaze-evoked orbital pain, active (inflammatory phase) TAO-related eyelid swelling, eyelid erythema, active (inflammatory phase) TAO-related conjunctival redness, chemosis, and inflammation of caruncle or plica, with a score ≥ 3/7 indicating active TAO as reported previously [14]. A-scan ultrasonography was used to determine the axial length (AL) (PacScan 300A). The average of five readings was used for each eye to avoid outlier readings. A standard deviation was 0.1 mm.

The spectral domain optical coherence tomography (SD-OCT) was used to perform the OCT (retinascan RS-3000 advance; NIDEK, Gamagori, Japan). For macular thickness and RNFL measurement, mydriasis was used to prepare the eyes using tropicamide 0.5% eye drops. Our findings were represented by a macular map, which consisted of nine sectorial thickness measurements arranged in three concentric circles with sizes of 1, 3, and 6 mm, making three rings. The superior, nasal, inferior, and temporal quadrants were separated into the two outer rings. RNFL thickness was measured using the Disc Map Protocol (6 × 6 mm). The previously described procedure was performed by the same experienced operator for all patients.

Electrophysiological tests were assessed with the RETI port/scan 21; (Roland Consult, Brandenburg, Germany). The parameters of the pattern visual evoked potential (PVEP) test and the recordings of the multifocal electroretinogram (mfERG) were in accordance with the International Society for Clinical Electrophysiology of Vision (ISCEV) standard [15, 16]. Also, PERG was performed according to Holder et al., 2007 [17].

### Statistical analysis

The Statistica software, version 12, was used to analyze the data. The mean and standard deviation (SD) of quantitative variables were calculated. The chi-square (*χ*2) was used to assess gender difference between the two studied groups. The data were statistically analyzed using an independent sample *t* test. The different variables that can affect the macular thickness were assessed using regression analysis and Pearson's correlation analysis. The significance of the results was determined at *p* < 0.05 levels.

## Results

In this study, a total of 80 eyes of 80 subjects were included, 40 in each group. They belonged to 29 males and 11 females without a statistically significant difference in the two studied groups. The male/female ratio was 2.3:1 in the control group and 3:1 in TAO group (*χ*2 = 0.25 and *p* = 0.62). The age range of patients group was between 25 and 72 years old (mean ± SD = 46.35 ± 13.32), which was age and gender matched with the control group (mean ± SD = 46.63 ± 13.63). Table 1 demonstrates the baseline demographic and ocular characteristics of both groups. There were no statistically significant differences between the two groups regarding age, spherical equivalent (SE), intraocular pressure (IOP), or AL, only the BCVA was significantly worse in the study group (logMAR 0.20 ± 0.24 vs. 0 ± 0, *p* < 0.001).

**Table 1:** Demographics in controls and patients with TAO

Variable	Control group	Patients with TAO	*t*-value	*p*
	Mean ± SD	Mean ± SD		
Age (years)	46.63 ± 13.63	46.35 ± 13.32	0.09	0.09
BCVA (logMAR) [range of Snellen acuities ratios]	0.00 ± 0.00 [20/20- 20/20]	0.20 ± 0.24 [20/40- 20/20]	− 5.23	< 0.001
SE (Diopters)	− 0.28 ± 0.38	− 0.29 ± 0.22	0.18	0.86
AL (mm)	23.16 ± 0.77	23.01 ± 0.75	0.89	0.37
IOP (mm Hg)	17.30 ± 2.88	16.60 ± 4.74	0.80	0.43

*Mean ± SD* mean ± standard deviation, *TAO* thyroid-associated ophthalmopathy, *BCVA* best-corrected visual acuity, *AL* axial length, *SE* spherical equivalent, *IOP* intraocular pressure

Slit lamp and fundus examination showed no abnormalities in either group. The degree of proptosis in TAO patients had a mean of 21.25 ± 3.04 mm (range, 18–28). The mean disease duration was 1.63 ± 0.70 years (range, 1–3 years) and the clinical severity score ranged from 2 to 5.

The results of OCT analysis are shown in Table 2. TAO patients had significantly decreased CFT and mean inner macular ring thickness, but there was no significant difference in mean outer macular thickness.

**Table 2:** Optical coherence tomographic measurements of macular retinal thickness of the controls and patients with TAO

Variable (µm)	Control group	Patients with TAO	*t*-value	*p*
	Mean ± SD	Mean ± SD		
CFT	257.35 ± 16.97	247.53 ± 9.63	3.19	0.002
SIM	349.80 ± 14.39	340.83 ± 8.73	3.37	0.001
IIM	329.80 ± 7.46	325.25 ± 6.26	2.95	0.004
TIM	330.40 ± 5.38	323.75 ± 10.08	3.68	< 0.001
NIM	320.20 ± 8.85	312.10 ± 12.63	3.32	0.001
Mean inner macular ring	332.49 ± 6.59	325.48 ± 5.03	5.35	< 0.001
SOM	298.75 ± 7.86	293.88 ± 8.63	2.64	< 0.001
IOM	283.30 ± 9.81	276.80 ± 7.38	3.35	0.001
TOM	265.48 ± 7.57	258.50 ± 8.96	3.76	< 0.001
NOM	301.03 ± 21.44	291.98 ± 13.81	2.24	0.03
Mean outer macular ring	287.16 ± 5.52	285.88 ± 5.22	1.07	0.29

*CFT* Central foveal thickness, *SIM* Superior of inner macular ring, *SOM* Superior of outer macular ring, *IIM* Inferior of inner macular ring, *IOM* Inferior of outer macular ring, *NIM* Nasal of inner macular ring, NOM Nasal of outer macular ring, TIM Temporal of macular ring, *TOM* Temporal of outer macular ring

All measured parameters of PERG and PVEP did not significantly differ between the two groups (Table 3), except for P50 amplitude which was significantly diminished in study group (2.82 ± 0.39 µV vs. 2.73 ± 0.42 µV, *p* = 0.02). Regarding the results of mfERG analysis (Table 4), there were no significant differences between the two groups in the retinal response density of ring 2, 3, 4 and 5 (R2, R3, R4, R5), however there were significant differences in the retinal response density of ring 1. TAO patients had significantly diminished ring1 P1 amplitude and diminished N1 amplitudes in the five rings, but R2, R3, R4, and R5 P1 amplitudes were insignificantly different between the two groups. In terms of the five rings latencies, there were no significant difference between the two groups.

**Table 3:** Pattern electroretinogram (PERG) and pattern visual evoked potential (PVEP) parameters in the studied groups

Variable	Control group	Patients with TAO	*t*-value	*p*
	Mean ± SD	Mean ± SD		
*PERG parameters*				
P50 implicit time (ms)	52.68 ± 0.63	52.72 ± 0.74	− 0.26	0.80
N95 implicit time (ms)	88.93 ± 6.92	89.51 ± 4.89	− 0.44	0.66
N35-P50 amplitude (µV)	2.80 ± 0.57	2.56 ± 0.27	− 2.33	0.02
P50-N95 amplitude (µV)	2.73 ± 0.42	2.82 ± 0.39	− 1.05	0.30
*PVEP parameters*				
N75 latency (ms)	81.43 ± 6.21	83.01 ± 4.82	− 1.27	0.21
P100 latency (ms)	106.16 ± 2.86	107.34 ± 3.53	− 1.64	0.11
N75-P100 amplitude (µV)	12.88 ± 0.94	13.26 ± 0.89	− 1.81	0.07

*µV* microvolts, *ms* milliseconds

**Table 4:** Comparison of multifocal electroretinographic five rings P1 and N1 amplitudes and latencies between the two studied groups

Variable	Control group	Patients with TAO	*t*-value	*p*
	Mean ± SD	Mean ± SD		
RRD1	48.93 ± 1.84	46.61 ± 6.51	2.17	0.03
RRD2	28.94 ± 0.95	27.67 ± 5.14	1.54	0.13
RRD3	21.30 ± 1.08	20.75 ± 1.96	1.55	0.13
RRD4	17.07 ± 1.32	16.77 ± 0.66	1.32	0.19
RRD5	12.79 ± 0.93	12.36 ± 1.003	1.98	0.05
R1 P1 amplitude (µV)	0.99 ± 0.18	0.91 ± 0.11	2.44	0.02
R2 P1 amplitude (µV)	0.89 ± 0.02	0.88 ± 0.05	1.27	0.21
R 3 P1 amplitude (µV)	0.68 ± 0.06	0.66 ± 0.04	1.33	0.19
R 4 P1 amplitude (µV)	0.63 ± 0.08	0.60 ± 0.10	1.64	0.10
R 5 P1 amplitude (µV)	0.59 ± 0.09	0.56 ± 0.06	1.40	0.17
Ring 1 P1 latency (ms)	41.31 ± 0.59	41.61 ± 0.87	− 1.77	0.08
Ring 2 P1 latency (ms)	41.14 ± 0.74	41.46 ± 0.84	− 1.82	0.07
Ring 3 P1 latency (ms)	41.54 ± 0.81	41.73 ± 1.15	− 0.85	0.40
Ring 4 P1 latency (ms)	42.67 ± 1.03	42.98 ± 0.92	− 1.41	0.16
Ring 5 P1 latency (ms)	43.29 ± 0.60	43.59 ± 1.02	− 1.59	0.12
Ring1 N1 amplitude (μV)	0.36 ± 0.02	0.34 ± 0.01	5.72	< 0.001
Ring 2 N1 amplitude (µV)	0.28 ± 0.01	0.26 ± 0.02	4.78	< 0.001
Ring 3 N1 amplitude (µV)	0.23 ± 0.02	0.22 ± 0.01	3.16	0.002
Ring 4 N1	0.19 ± 0.01	0.18 ± 0.02	2.11	0.04
Ring 5 N1 amplitude (μV)	0.13 ± 0.02	0.12 ± 0.02	2.12	0.04
Ring 1 N1 latency (ms)	22.88 ± 1.13	22.73 ± 0.97	0.64	0.53
Ring 2 N1 latency (ms)	23.96 ± 1.23	23.86 ± 1.03	0.39	0.70
Ring 3 N1 latency (ms)	24.42 ± 1.35	24.27 ± 1.32	0.50	0.62
Ring 4 N1 latency (ms)	24.32 ± 1.28	24.59 ± 0.88	1.12	0.27
Ring 5 N1 latency (ms)	24.63 ± 1.62	24.93 ± 1.12	0.96	0.34

*TAO* Thyroid-associated orbitopathy, *µV* microvolts, *ms* Milliseconds

When studying correlation (Table 5), CFT of TAO patients showed a significant positive correlation with BCVA, P50 amplitude, and R1 N1 amplitude, while exhibited a negative correlation with disease duration, proptosis degree, clinical activity score, and R1 N1 delay. However, there was no significant relationship between age, SE, AL, CCT, RRD1, ring1 P1 amplitude, and ring1 P1 latency. On multiple regression analysis (Table 6), we found that degree of proptosis and P50 amplitude were the most important determinants for CFT. Central foveal thickness was not affected by age, BCVA, SE, AL, IOP, CCT, RRD1, duration of disease, ring1 P1 amplitude, ring1 P1 latency, ring1 N1 amplitude, ring1 N1 latency or CAS.

**Table 5:** Correlations between CFT and different parameters in the patients with proptosis

Variables	Cases with proptosis	
	Central foveal thickness	
	*r*	*p*
Age (years)	− 0.17	0.29
BCVA (logMAR)	− 0.64	< 0.0001
BCVA (range of Snellen acuities ratios)	0.65	< 0.0001
SE (diopters)	0.03	0.85
AL (mm)	− 0.12	0.46
IOP (mm Hg)	− 0.24	0.14
CCT (mm)	0.18	0.26
Degree of proptosis	− 0.59	< 0.0001
duration of the disease (years)	− 0.78	< 0.0001
RRD1 (nV/deg^2^)	− 0.07	0.67
Ring1 P1 amplitude (µV)	0.22	0.17
Ring1 P1 latency (ms)	0.05	0.76
Ring1 N1 amplitude (µV)	0.51	< 0.0001
Ring1 N1 latency (ms)	− 0.41	< 0.0001
CAS	− 0.90	< 0.0001

*BCVA* best-corrected visual acuity, *logMAR* logarithm of the minimal angle of resolution, SE spherical equivalent, *AL* axial length, *IOP* intraocular pressure, *CCT* central corneal thickness

**Table 6:** Multiple regression analysis of different factors affecting CFT in in the patients with TAO

	Beta	B	*t*	*p*-level
Intercept		37.02	0.18	0.86
Age (years)	− 0.22	− 0.16	− 1.78	0.09
BCVA (logMAR)	− 0.28	− 10.06	− 1.22	0.24
SE (Diopters)	0.16	7.00	1.24	0.23
AL (mm)	− 0.18	− 2.32	− 1.201	0.24
IOP (mm Hg)	− 0.06	− 0.13	− 0.36	0.72
Degree of proptosis in mm	− 0.56	− 1.76	− 2.29	0.03
Duration of the disease (years)	0.61	8.47	1.39	0.18
RRD 1 (nV/deg^2^)	− 0.10	− 0.15	− 0.62	0.54
P50 implicit time (ms)	− 0.14	− 1.86	− 0.95	0.35
N35 − P50 amplitude (µV)	0.39	6.51	2.59	0.01
Ring1 P1 amplitude (µV)	0.13	11.57	0.92	0.37
Ring 1 Latency (ms)	− 0.02	− 0.21	− 0.12	0.91
Ring1 N1 amplitude (µV)	0.53	422.01	1.01	0.33
Ring1 N1 Latency (ms)	0.69	6.91	1.51	0.15
Clinical activity score	− 0.62	− 5.27	− 1.09	0.29

*BCVA* best-corrected visual acuity, *logMAR* logarithm of the minimal angle of resolution, *SE* spherical equivalent, *AL* axial length, *IOP* intraocular pressure

Regression summary for central foveal thickness of TAO patient *R* = 0.82 *R*^2^ = 0.67 Adjusted *R*^2^ = 0.61 *F* (14, 85) = 12.21 *p*

On multiple regression analysis, we found that degree of proptosis and P50 amplitude were the most important determinants for CFT. Table 6

## Discussion

In this work, we demonstrated the successful use of OCT and EPS in the evaluation of proptotic eyes of patients with TAO and correlated the findings to their clinical presentation.

We found that the CFT and mean inner macular ring thickness were significantly thinner in proptotic eyes, although mean outer macular thickness exhibited no significant changes. This is consistent with the findings of Meirovitch et al. [18], who showed that TAO patients' eyes had a considerable thinning of the inner macula when compared to healthy control eyes. In addition, Sayn et al. [10] stated that TAO patients' macular thickness was thinner in comparison with their controls. Casini et al. [19] discovered that in Graves' orbitopathy patients, central retinal thickness, and central ganglion cell complex (GCL) thickness were lower than in patients than controls.

Macular thinning in our TAO eyes could be caused by orbital contents compressing the retina, or it could be a common sign in people with autoimmune retinopathy due to damage induced by anti-retinal antibodies [20]. Casini et al. [19] demonstrated that Graves' orbitopathy cases had significant alterations in foveal and GCL thickness, as well as optic nerve head morphology, implying that the orbital inflammatory process may play a role. An alternative possible cause of macular thinning in TAO patients is reduction in blood supply to the retina. According to Fernandez-Buenaga et al. [21] macular thinning was found in the patients with non-arteritic anterior ischemic optic neuropathy (NAION), which could be explained by ischemic damage in the maculopapillary bundle that occurs in NAION. The decrease in blood flow in TAO disease may contribute to macular thinning via vein congestion or arterial stenosis. This can explain the foveal and retinal function impairment in our TAO eyes which can be attributed to long-lasting chronic impairment of blood supply of the eyeball [4].

Previous studies on TAO have addressed the impact of proptosis on various electrophysiologic studies. While multiple studies utilized VEP [22–25] and PERG [26–28], to the best of our knowledge this is the first study to use mfERG to assess the disease, and to correlate this alteration with OCT findings to explain vision loss in patients without optic neuropathy.

Despite the absence of visible retinal abnormalities in the TAO group, they had lower mean BCVA when compared to controls, and mfERG response densities (RRD) showed a considerably lower value of ring1 than controls. Furthermore, in the cases, PERG P50 had a statistically significant lower amplitude. This may indicate early macular insult in these patients.

We noted that the four-quadrant N1 amplitude is smaller, despite the fact that there was no statistical difference between the two groups when it came to the mean quadrant N1 amplitude. It is worth noting that the electrophysiologic tests as mfERG and P50 wave of PERG, which assess macular function, revealed substantial differences between patients and controls. While PVEP and the N95 wave of the PERG, which test visual pathway and optic nerve function, found no statistically significant differences between the groups. These findings suggest that TAO may have an impact on retinal and macular function.

Retinal atrophy and lower macular thickness on OCT with corresponding diminished central response on mfERG were tested by Abazari et al. [21] similar alterations were deemed common in people with autoimmune retinopathy. This could help researchers figure out how visual impairment occurs and how to diagnose autoimmune retinopathy.

According to Meirovitch et al. [19], OCT abnormalities imply that the retina is involved in TAO, as early as the preclinical stage, and that early detection of retinal abnormalities could avert serious visual consequences. Multiple regression models were used to determine which component was the most important determinant of CFT in TAO patients. We found that the degree of proptosis was the most important determinant for CFT. From previous findings of earlier retinal involvement than optic nerve in TAO, a specific retinal involvement which can be autoimmune in nature not just a mechanical effect may be suspected.

Our study has various limitations, the most significant of which is the small sample size multicenter studies may be more valuable, and a cross-sectional design would not provide more thorough data. Antithyroid antibody testing was not undertaken in our cohort, with sole reliance on serum thyroid hormonal levels and clinical examination for confirmation of the diagnosis. Finally, evaluation of the retinal microvasculature and their association with clinical and electrophysiologic variables was not conducted. A future study utilizing optical coherence tomography-angiography in TAO patients to investigate such subject is currently underway.

## Conclusion

Macular thinning along with functional alterations noticed by PERG and mfERG could be associated with subclinical retinopathy in TAO cases in the absence of fundus abnormalities.
